# Editorial to “Incidence of nausea/vomiting following propofol sedation with adaptive‐servo ventilation for atrial fibrillation ablation”

**DOI:** 10.1002/joa3.13024

**Published:** 2024-03-21

**Authors:** Mark T. Mills, Peter Calvert, Dhiraj Gupta

**Affiliations:** ^1^ Liverpool Centre for Cardiovascular Science at University of Liverpool, Liverpool John Moores University and Liverpool Heart & Chest Hospital Liverpool UK; ^2^ Department of Cardiology Liverpool Heart & Chest Hospital NHS Foundation Trust Liverpool UK

**Keywords:** Atrial fibrillation, Catheter ablation, Vagal injury, Nausea and vomiting

## Abstract

The incidence of nausea, vomiting, and symptoms relating to vagal nerve injury remains high after atrial fibrillation ablation, with many patients reporting symptoms in the hours to months after their procedure. These are often underreported in literature, and this editorial piece opines about a study assessing this in detail.

Catheter ablation for atrial fibrillation (AF) can be performed under general anesthesia, deep sedation, or mild conscious sedation, with purported benefits of each approach. When considering AF ablation, clinicians and patients must carefully balance the potential for symptom improvement against procedural risks, complications, and adverse effects. Complications such as groin bleeding, tamponade, and stroke are well recognized and therefore carefully explained to patients prior to their procedure. However, the incidence of more subtle adverse effects—such as nausea, vomiting, and gut dysmotility—are less well understood, and as a result, less well explained. Such adverse outcomes, although often transient, can last weeks to months, or occasionally cause long‐term distress. Gaining a better understanding of these effects is critical to ensuring patients are fully informed of potential side effects prior to ablation, and to enable clinicians to minimize risk and provide appropriate treatment. In this issue of the *Journal of Arrhythmia*, Sakanoue and colleagues^1^ provide insightful new data into this scarce field, which we explore in this editorial.

## 1 POSTOPERATIVE NAUSEA AND VOMITING

Postoperative nausea and vomiting (PONV) is one of the most common side effects after anesthesia, occurring in up to a third of patients.^2^ Many aspects contribute toward the development of PONV, including patient‐related (e.g., biological sex and prior history of PONV), anesthesia‐related (e.g., type of anesthesia and specific agents used), procedure‐related (e.g., duration and location of procedure), pain‐related (e.g., uncontrolled pain), and pharmacological (e.g., use of opiates) factors.^2^

In the present study, Sakanoue et al. retrospectively assess the incidence of PONV in 272 patients undergoing AF ablation.^1^ Of note, all procedures were performed according to a standardized institutional protocol, which included the use of radiofrequency ablation (30–35 watts, 20–30 second lesions, without esophageal temperature monitoring), deep sedation with propofol, and adaptive servo‐ventilation (ASV). ASV, a form of positive airway pressure therapy similar to continuous and bilevel positive airway pressure therapies, has previously been demonstrated as advantageous over traditional ventilation methods, resulting in fewer restless body movements and improved ablation parameters.^3^ Abiding by this protocol, the incidence of PONV within 12 h of ablation was 5.5%. Interestingly, the Apfel score, a previously validated predictor of PONV, performed only modestly, with an area under the receiver operating characteristic curve of 0.66. Other than a prior history of motion sickness or PONV, which was more common in those experiencing PONV, all other documented characteristics—such as demographics, peri‐procedural opiate and sympathomimetic use, and procedural duration—were similar between those with and without PONV. The authors contrasted their findings with the published literature, citing a wide incidence of PONV from 4.5% to 78.5%, and concluded that their standardized protocol was associated with a favorable side‐effect profile.

The authors should be congratulated for these much‐needed figures on the incidence of PONV following AF ablation, and for providing further evidence in support of ablation under deep sedation. However, we must acknowledge the limitations of their study. Most importantly, this was a single‐center, retrospective, observational study, with no control group. While helpful in assessing the incidence of PONV using their defined protocol, direct comparison of these results with those of other centers and other protocols, such as general anesthesia or mild conscious sedation, is not possible. Second, patients were only monitored for nausea and vomiting in the immediate (12‐h) postablation period; unfortunately, no information on subsequent symptom resolution or persistence is provided, nor is the rate of peri‐procedural anti‐emetic use among either group. This limits extrapolation of these findings to the postprocedural period, during which patients are generally discharged from the hospital, but may experience ongoing or new symptoms without seeking medical advice.

## 2 ABLATION‐INDUCED GASTROINTESTINAL DYSMOTILITY

Beyond PONV, which can occur following any procedure under anesthesia, AF ablation itself is known to be associated with postprocedural nausea, vomiting, and gastrointestinal dysmotility,^4^ irrespective of the anesthetic or sedation method used. Vagal nerve injury as a result of thermal ablation is commonly cited as the etiological mechanism for this phenomenon. Indeed, in studies that carefully document the incidence of vagal nerve symptomatology following AF ablation, the incidence of moderate nausea or vomiting at 1‐month postablation is 17% and 7.5%, respectively, with severe nausea and vomiting in 8% and 1.5%. This is in addition to around a quarter to a third of patients who experience issues with heartburn, diarrhea, and early satiety.^4^

Although these symptoms generally resolve by 3 months^4^—interestingly, the timepoint at which patients usually undergo their first postablation medical review—it is imperative for electrophysiologists to recognize the high incidence of these symptoms, educate our patients during pre‐ablation consultations, and advise on postablation strategies to alleviate these effects, for example, the prescription of proton‐pump inhibitors and anti‐emetics. With the advent of pulsed‐field ablation—with reported advantages in terms of cardioselectivity—it remains to be determined whether the incidence of ablation‐induced gastrointestinal dysmotility is reduced.

## 3 DEEP SEDATION FOR AF ABLATION

Finally, the study by Sakanoue and colleagues^1^ demonstrates the feasibility and safety of AF ablation under deep sedation, in the absence of complications or conversion to general anesthesia. Although general anesthesia is often used as a standard for AF ablation in privatized healthcare systems, its availability in publicly funded systems can be limited, because of cost and workforce planning issues. In the present study, deep sedation was delivered by a specialized nurse, under supervision of the electrophysiologist, and without an anesthetist present.^1^ However, legal requirements for the performance of deep sedation continue to vary worldwide, with a recent survey highlighting that 59.6% of centers continue to mandate the presence of an anesthetist for this approach.^5^ The findings from the present study, and others like it, are further endorsement for the adoption of AF ablation under deep sedation, and should be considered by regulatory institutions across countries and centers, given the rising number of patients eligible for—and likely to benefit from—AF ablation.

## 4 CONCLUSION

Unfortunately, little consideration is often given to nausea and vomiting following catheter ablation of AF, perhaps as it considered a relatively minor side effect of this effective, guideline‐directed treatment for symptomatic AF. Despite this, the incidence of nausea, vomiting, and symptoms relating to vagal nerve injury remains high after ablation, with many patients reporting symptoms in the hours to months after their procedure. Deep sedation, the development of technologies that reduce the likelihood of gastrointestinal dysmotility, and the prompt recognition and treatment of symptoms are important strategies in improving the experience of patients during their postablation recovery.

## FUNDING INFORMATION

None.

## CONFLICT OF INTEREST STATEMENT

MTM and PC have no conflicts of interest to declare. DG reports institutional research grants from Boston Scientific and Medtronic, and speaker fees from Boston Scientific.

## ETHICS STATEMENT

None.
